# Video-Sensing Characterization for Hydrodynamic Features: Particle Tracking-Based Algorithm Supported by a Machine Learning Approach

**DOI:** 10.3390/s21124197

**Published:** 2021-06-18

**Authors:** Aimé Lay-Ekuakille, John Djungha Okitadiowo, Moïse Avoci Ugwiri, Sabino Maggi, Rita Masciale, Giuseppe Passarella

**Affiliations:** 1 Department of Innovation Engineering, University of Salento, 73100 Lecce, Italy; 2 Department of Information Engineering, Infrastructure and Sustainable Energy (DIIES), University “Mediterranean” of Reggio Calabria, 89124 Reggio Calabria, Italy; johnpdjungha@unirc.it; 3 Department of Industrial Engineering, University of Salerno, 84084 Fisciano, Italy; mavociugwiri@unisa.it; 4 CNR, National Research Council, Institute of Atmospheric Pollution Research, 70126 Bari, Italy; sabino.maggi@cnr.it; 5 Faculty of Engineering, International Telematic University UniNettuno, 00186 Rome, Italy; 6 CNR, National Research Council, Water Research Institute, 70132 Bari, Italy; rita.masciale@ba.irsa.cnr.it (R.M.); giuseppe.passarella@ba.irsa.cnr.it (G.P.)

**Keywords:** sensors, sensing systems, hydrodynamic monitoring, flow measurement and classification, machine learning, particle tracking

## Abstract

The efficient and reliable monitoring of the flow of water in open channels provides useful information for preventing water slow-downs due to the deposition of materials within the bed of the channel, which might lead to critical floods. A reliable monitoring system can thus help to protect properties and, in the most critical cases, save lives. A sensing system capable of monitoring the flow conditions and the possible geo-environmental constraints within a channel can operate using still images or video imaging. The latter approach better supports the above two features, but the acquisition of still images can display a better accuracy. To increase the accuracy of the video imaging approach, we propose an improved particle tracking algorithm for flow hydrodynamics supported by a machine learning approach based on a convolutional neural network-evolutionary fuzzy integral (CNN-EFI), with a sub-comparison performed by multi-layer perceptron (MLP). Both algorithms have been applied to process the video signals captured from a CMOS camera, which monitors the water flow of a channel that collects rain water from an upstream area to discharge it into the sea. The channel plays a key role in avoiding upstream floods that might pose a serious threat to the neighboring infrastructures and population. This combined approach displays reliable results in the field of environmental and hydrodynamic safety.

## 1. Introduction

Discharge channels are artificial or natural, intermittent or perennial channels functionally used to drain marsh areas or to collect rain waters. They convert a widespread water flow into a somewhat regular flow. The flow measurement in an open channel, as well as onsite dynamic conditions, can be carried out using diverse instruments and devices, such as flowmeters, and a panoply of sensors. We can use different principles for these instruments, notably ultrasonic, hydrostatic, and lasers. For small channels, laser flowmeters are mostly used, given the fact they utilize either single or multiple points for measuring velocities under the water surface. For water flow containing materials, to get accurate measurements despite solid materials, sensors based on the bubble level are suitable. The flowmeter based on an electromagnetic current also falls within the materials in the channel.

The key principle of the ultrasonic, radar, or pressure device is related to the indirect measurement, that is, the measurement of the flow (*Q*) is given by the water speed (*V*) to be multiplied by the area (*A*) of the channel. Then, the flow is explained by the continuity equation, namely Equation (1)

(1) Q=VA

This relationship is an indirect measurement, and the uncertainty is caused by the quality of the instrument and the linearity of the channel. The maintenance aspects connected to the instruments and devices are the main constraints for the limitations of this hardware, especially in areas where it is difficult to protect it. A compromise can be found by using a minimum hardware with a good quality of processing to monitor the scene. Certainly, the use of camera sensors, installed on a pole with the camera eye directed to the flow within the channel, could be the correct compromise, but there would need to be strong treatment of the captured signals.

For unsupervised channels located in isolated areas, low-cost hardware should be adopted for the instruments and devices, namely camera sensors, but with the help of excellent processing techniques so as to compensate for the low-cost hardware performance. Traditional image processing can be adopted, as is done in object/video recognition. A convolutional neural network (CNN) with algorithm branches can be useful for performing video recognition and classification. The water motion, along with the channel features, can be re-conducted for motion recognition issues, such as facial emotions recognition and the recognition of walking people, which are faced by some branches of CNNs, notably multiple convolutional neural networks employing improved fuzzy integral (MCNN-IFI). This article proposes an alternate method for a CNN branch connected to an evolutionary fuzzy integral (EFI), and its advantages are reported in the appropriate sections.

The proposed suite of tools can be easily implemented using a smartphone as an image sensor, as these devices are capable of producing excellent results even in fixed station.

## 2. Material and Methods

**Figure 1:**
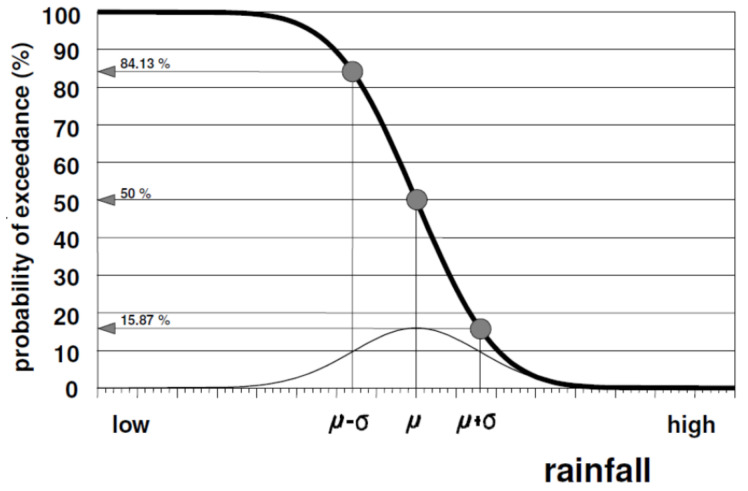
Exceedance probability as a function of rainfall.

The capability of collecting rain water is connected to the probability of the occurrence of a rainfall depth greater than some given value X_P_, denoted as the probability of exceedance P, which is expressed either as a fraction (on a scale ranging from 0 to 1) or as a percentage chance (on a scale ranging from 0 to 100%). Figure 1 shows the exceedance probability [1] as a function of the rainfall.

The evaluation of the overflow is thus fully related to the aforementioned idea. If we consider a basin of area *A* with a height of rainfall *h* (covering the area *A*), the volume of water that flows to the network is given by the following equation

(2) V=KhA

where K is the coefficient of the ponderal and the average discharge of the basin. Here, *h* is usually expressed in mm and *A* is in hectares. To express *V* in m^3^, we need to convert all of the variables into SI units, obtaining the following

(3) V=10KhA

The average flow of the general section *S* subtended by the basin of area *A* is given by the following expression

(4) $$Q=\frac{V}{T_{r}+T_{c}}=\frac{10KhA}{T_{r}+T_{c}}$$

where *T_r_* (rainfall time) [2] and *T_c_* (corrivation time) [3], or the time of concentration, are expressed in days, and the flow Q is in m^3^/day. The above approach is defined as a kinematic method. There are some uncertainties in the assessment of the corrivation time, which can be expressed using different expressions, such as those of Turazza [4], Ventura [5], and Pasini [6]. For natural hydrographic networks, we used the well-known Giandotti’s formula

(5) $$T_{c}=\frac{a\sqrt{A}+bL}{c\sqrt{H}}$$

where *T_c_* is expressed in hours, *A* in km^2^, *L* is the maximum distance travelled by water in km, and *H* is the average height of the basin in m. The coefficients of Equation (4) are set as follows: a = 4, b = 1.5, and c = 0.8. In the limit of *T_r_ = T_c_*, Equation (3) becomes the following

(6) $$Q_{\max}=\frac{\lambda Ah\sqrt{H}}{4\sqrt{A}+1.5L}$$

where *h* is expressed in m (height of rainfall). The coefficient *λ* is inversely proportional to the surface of the basin *A* and varies from 166 to 66 for *A,* ranging from 500 to 70,000 km^2^.

Setting *T_r_ = T_c_* implies that the maximum flow is reached immediately. In this particular case, *Q = V/2T_c_* and *Q_max_ = V/T_c_*, demonstrating that the maximum flow is twice that of the average flow. For small basins, Equation (5) underestimates the real values of *Q_max_*.

Given the considerations above, a monitoring system based on still images cannot be accurate without an automatic measurement of the real depth of the channel. Conversely, a video-sensing system with camera-grade sensors could be a viable and accurate alternative, because processing video recorded scenes allows for extracting interesting features of water flow by means of machine learning techniques and subsequent classification.

## 3. Study Area and Local Context

**Figure 2:**
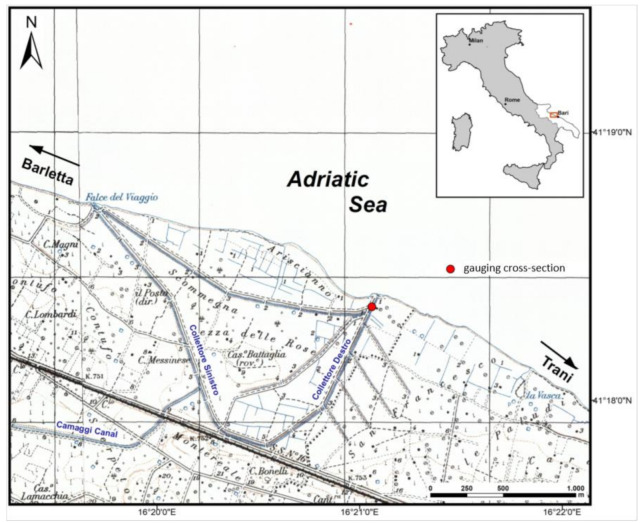
Study area and location of the drainage channels and gauging cross-sections.

The study area is located in the Apulia Region, in Southern Italy. The study reported in this paper focuses in particular on the Collettore Destro, an artificial spring drainage channel that is the most sizable among a group of channels located along the coastal belt between the towns of Barletta and Trani (Figure 2).

**Figure 3:**
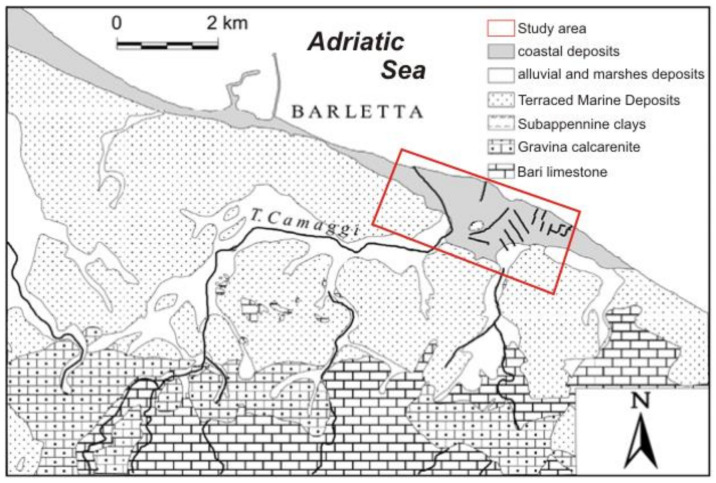
Geological setup of the area (modified after Caldara et al., 1998 [8]).

From a geological point of view, the territory surrounding the city of Barletta consists of several units overlapping each other. As Figure 3 clearly shows, moving up from the bottom to the top, these units consist of the limestone of Bari (Cretaceous); the calcarenite of Gravina (upper Pliocene–lower Pleistocene); the Subapennine clays (lower Pleistocene); the Terraced Marine Deposits (middle–upper Pleistocene); and lastly, the alluvial, marshes, and coastal deposits (Holocene) [7].

The study area can be considered to be a transition zone between two important structural domains, namely: the Apulian foreland to SSE and that of the Apennine foredeep to NNW. From a tectonic point of view, this area was considered relatively stable until a few decades ago. However, Ciaranfi et al. [7] showed that Quaternary tectonic movements have affected this area, dislaying a decisive influence on the actual geomorphological and hydrogeological structure. Disjunctive type tectonic faults dislocate the Cretaceous bedrock, creating a horst and graben setting oriented towards the Adriatic coast and the Ofanto Valley. This geological setting makes the presence of two specific aquifer structures possible: the deep carbonate aquifer (which is the primary aquifer), permeable by fracturing and karst phenomena, and the shallow porous aquifer, corresponding to sand-calcarenite levels of terraced marine deposits.

In the carbonate aquifer, freshwater floats over saltwater not only in the coastal zone, but also inland. Here, the freshwater/saltwater interface occurs thousands of meters below sea level, because of the high value of the hydraulic head [9]. In particular, in this area, the disjunctive structures cause the presence, at different depths below the sea level, of thick dolomitic layers that, having low permeability, cause the carbonate aquifer to be under pressure (confined) and the aquifer roof to be always below sea level (about 200–400 m). Consequently, the freshwater/saltwater interface is located at great depths, even in close proximity to the coast. The main outflow of groundwater in this aquifer portion is represented by the group of springs emerging along the coastal zone.

The study area is characterized by a dry–sub-humid, Mediterranean climate, typical of the whole Apulia region. Precipitation is scarce, around 400 mm/year on average, and temperatures are mild over most of the year. Summers are long, dry, and hot, summer temperatures often exceed 40 °C, and rainfall can be absent for two or three consecutive months. In winter, precipitation is scarce and irregular, and temperatures are mild, usually around 10 °C [10,11,12,13].

From an environmental point of view, this area is a widespread wetland (Ariscianne–Boccadoro wetland), characterized in the past by the presence of marshes formed as a result of the numerous springs draining not only the carbonate aquifer, but also the shallow porous one. The marsh reclamation began at the beginning of the 19th century, but the ultimate reclamation took place in 1939 when a network of drainage channels was built to allow for the outflow of stagnant water. The current morphology of the area is flat with a slight slope towards the sea and altitudes up to 5 m above sea level. During the reclamation works, the flow of the Camaggi torrent was regulated within an artificial channel. Moreover, following the natural slopes of the surface, several draining channels were created in its final section, forming the Collettore Sinistro to the West and the Collettore Destro to the East.

**Figure 4:**
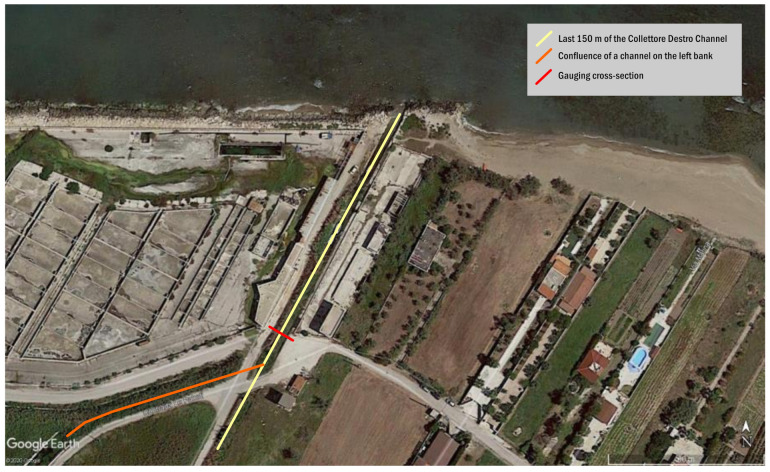
Aerial picture of the study area showing the last stretch of the “Collettore Destro” drainage channel and the location of the gauging cross-section during the monitoring of 2007–2011.

In particular, the Collettore Destro is composed of several drainage channels that merge into a single channel before spilling into the sea. The channel’s gauging cross-section considered in this work is located downstream of the confluence (Figure 4).

The physico-chemical analysis carried out by the regional water authority on water samples taken at the source showed fixed residue values of about 3–4 g/L at 180 °C, therefore with a significant influence of saltwater intrusion.

**Figure 5:**
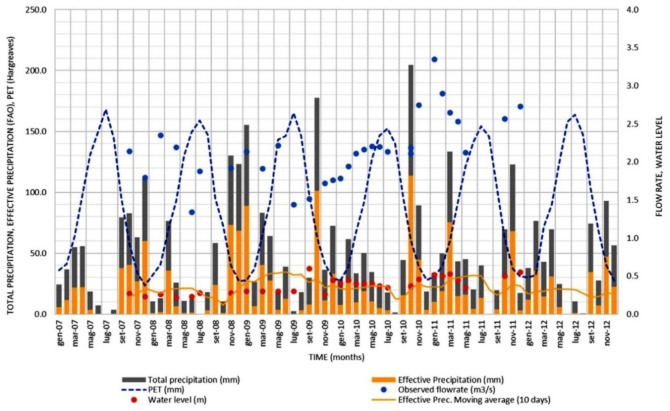
Elements of the water balance in the area under investigation and the measured flow rate and water level at the testing cross-section of the Collettore Destro channel. The effective precipitation and the potential evapotranspiration (PET) are assessed according to the Brouwer and Heibloem [14] and Hargreaves formulas, respectively.

Flow measurements for the Collettore Destro are available from the 1920s to the 1980s, although they are very discontinuous over time. The measured flow rate during this period was constant, being around 0.5 m^3^/s. During the first decade of the current century, within the framework of a regional monitoring project, occasional flow and water level measures were carried out in a section of the channel located about 100 m from its outlet into the sea. These surveys revealed very different flow rates from those of the last measures, dating back to about 35 years earlier, which reached values above 2.0 m^3^/s. This difference led to the need to proceed with further investigations [14]. Figure 5 shows some elements of the water balance in the area under investigation, together with the measured flow rate and water level at the testing cross-section of the Collettore Destro channel between 2007 and 2012.

The effective precipitation and the potential evapotranspiration (PET) were assessed based on [15] and the Hargreaves formula, respectively. As reported above, the average flow rate from 2007 to 2012 was about 2.0 m^3^/s, with a maximum flow rate of about 3.9 m^3^/s in January 2011 and a minimum of 1.3 m^3^/s in June 2008. The estimated PET was around 1000 mm/year, as confirmed by the measured values reported in the regional meteorological and climatic statistics published by the Ministry of Agriculture (https://www.politicheagricole.it/, accessed on 18 June 2021).

The precipitation and temperature data used to calculate the PET and the effective precipitation, shown in Figure 5, were downloaded by the Hydrological Annals published by the Apulian Civil Protection [16]. In particular, the time-series of the weather station of Andria, located a few kilometers away from the study area, were used for this study.

**Figure 6:**
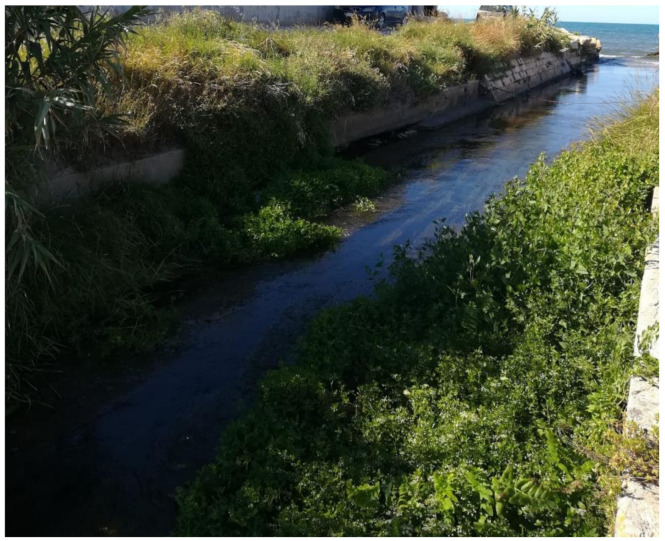
The drainage channel Collettore Destro at its outlet into the Adriatic Sea.

As clearly shown in the picture in Figure 6, shot during the summer of 2020, the channel is currently wrongly maintained, and reeds and small shrubs abound along its banks, while a carpet of algae overlays its bottom. This lack of proper maintenance not only complicates the measurement of the water level and flow and reduces its reliability, but, what is worse, prevents the natural flow of water to the sea, provoking recurring overflowing events upstream.

## 4. Proposed Algorithms and Methods

### 4.1. Particle Tracking

A fundamental question in motion tracking is whether it is possible to track a single selected object in an environment of multiple moving objects. A proper answer to this question could be beneficial to several different applications.

In particular, video sequence analysis is needed to track an object in each frame of a captured video. Many tracking methods have been investigated recently [17], including region-based tracking, contour-based tracking, feature-based tracking, and model-based tracking. In this work, we perform an automatic object motion-based tracking using a video taken with a stationary camera, analyzing the relative movement of water with respect to the grass.

**Figure 7:**
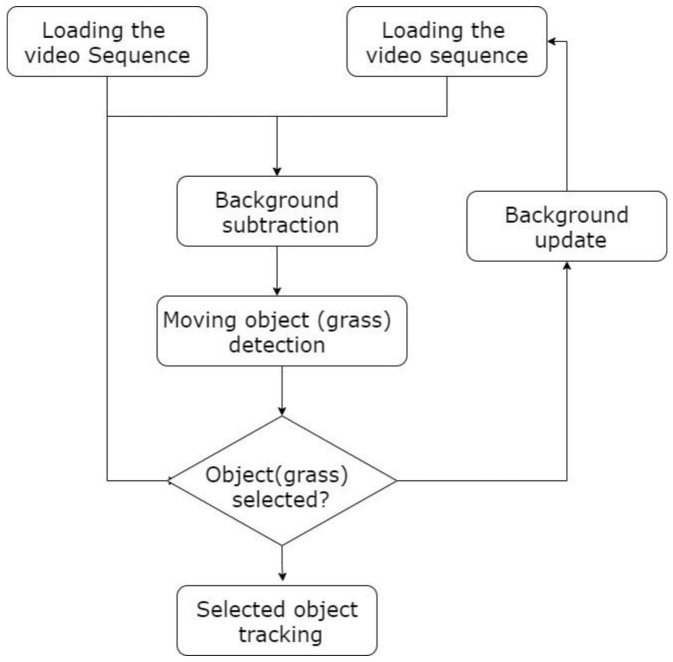
Flowchart of the algorithm used in this work.

The approach used in this work uses a background subtraction algorithm based on Gaussian mixture models [18]. Morphological operations were applied to the resulting foreground mask to remove the noise. Blob analysis was also applied to detect groups of connected pixels [19]. The particular filtering used here is based on a Monte Carlo approximation of the optimal Bayesian filter. In practice, this is achieved through the calculation of the posterior probability of a first-order Markov process, as shown in Equation (6).

(7) $$p(x_{1}|y_{1:t})=\alpha p(y_{t}|x_{t})\int_{x_{t-1}}p(x_{t}|x_{t-1}|y_{1:t-1})$$

where $x_{t}$ is the process state at time *t*, $y_{t}$ is the corresponding observation, and $y_{1:t-1}$ is the set of all observations across time *t*. The conditional probability $p(x_{1}|y_{1:t})$ is the dynamic distribution of the process, while $p(x_{t}|y_{t})$ is the observation likelihood distribution and α is a normalization factor. Figure 7 presents the flow chart of the algorithm used in this work.

The integral form of Equation (6) does not have a closed-form solution, but can be approximated using a set of weighted samples ${\left(x_{1}^{\left(i\right)},\pi_{1}^{i}\right)}_{i=1,\ldots ,n}$, where $x_{t}^{\left(i\right)}$ is the corresponding particle weight. Under this representation, the approximation to the Bayesian filtering formula becomes

(8) $$p(x_{t}|y_{1:t}\approx \alpha p(y_{t}|x_{t})\sum_{i=1}^{n}\pi_{t-1}^{\left(i\right)}p{\left(x_{t}\right)}^{\left(i\right)}|x_{t-1}^{\left(i\right)})$$

It is noteworthy to mention here that the implementation of the algorithm is based on the procedure used in the motion generic edge tokens (GETS) [20]. All new candidates of the generated cluster lying on the same trace were evaluated. If the size of the cluster was less than a predefined threshold, they were discarded as noise, otherwise the cluster was stored as a region. The procedure was repeated until all of the labelled moving particles in water were tracked. If the same region shared a similar GET stream pattern in two consecutive frames, it was assigned the same label. A specific performance metric was used to evaluate the accuracy of the tracker. Here, we used the generalized optimal subpattern assignment (GOSPA) metric, which evaluates the performance of the tracker by assigning it a single cost value. An improvement in the tracking behavior decreased the corresponding value of the GOSPA metric.

### 4.2. Machine Learning and Classification

**Figure 8:**
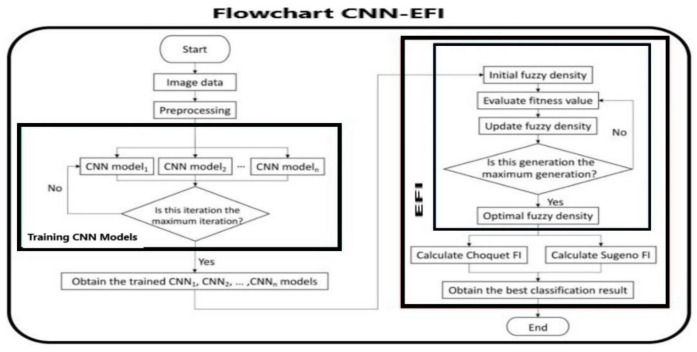
Flowchart of a convolutional neural network (CNN) including an evolutionary-fuzzy-integral routine.

To maximize the efficiency of the convolutional neural network (CNN) model, it is important to include other neural architectures and techniques, namely the evolutionary fuzzy integral (EFI) [21], as shown in the flowchart of Figure 8. An image pre-processing step is required before applying the CNN model, which takes into account the data used as the input of the proposed model and connected to the previous step, called background. In this way, the retrieved image allows to create and to complete the CNN model.

Depending on the good and appropriate fitness values, we chose the fuzzy density values for a good optimization; during this operation, using the storage of the classifier and a description of the result set, the goal is to deliver a result with a better and good precision. CNN provides the output that will be used as the inputs for the scalable Fuzzy integration.

Assuming $X={\left\{x_{i}\right\}}_{i=1,\ldots ,n}$ represents a set of *n* classifiers (in this work, *n* was set to 3), the fuzzy measure $g\left(\left\{x_{i}\right\}\right)$ can be thought as the value of the subset $\left\{x_{i}\right\}$ ranging between 0 and 1, as reported in Equation (8)

(9) $$if\;A\subset B\subset X\;then\;0\leq g\left(A\right)\leq g\left(B\right)\leq 1$$

When there is only one element in the set X, the fuzzy measure $g\left(X\right)$ is called the fuzzy density. Before calculating other fuzzy measures, the fuzzy density must first be defined. In conventional fuzzy integral applications, the fuzzy density is defined by users according to the particular problem to be solved.

**Figure 9:**
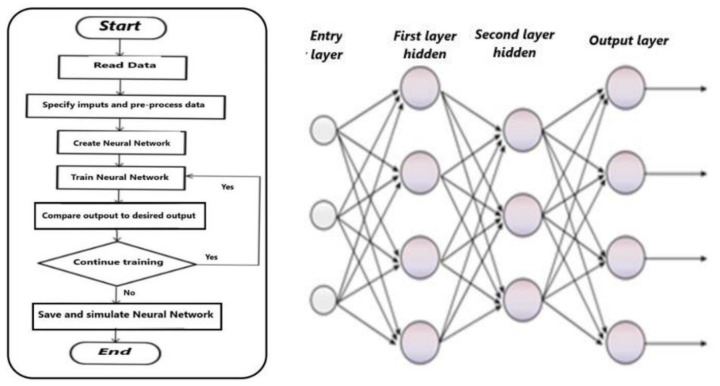
Flowchart of the proposed multi-layer perceptron (MLP) (**left**) and its internal structure (**right**).

Conversely, with its logic of circulating data only in one direction, i.e., from the input layers to the output layers, the multi-layer perceptron (MLP) is a neural network that flows information directly [22]. Each layer of neurons is fully connected to the neighboring layers. Within the multilayer backpropagation perceptron, the destination neurons experience an effect that alters their information. It must be noted that the neural network automatically finds the optimal values of the weights associated with each connection between the between the different layers of the network. A good connection between the neurons gives a better resolution according to the flowchart shown in Figure 9.

As reported in the introduction, IFI and EFI CNN-based branches are used in motion issues. Both IFI and EFI partly include a fuzzy approach supported by a convolutional direction. We know that fuzzy operations are symbolic and CNN ones are numerical. Hence, they encompass the advantages of symbolic and numerical treatments. Both IFI and EFI increase the classification accuracy if combined with CNN. However, EFI displays a significantly better classification rate along with a low computational time, especially for complex recognition, as in our case. In both architectures, CNN is applied prior to any fuzzy step. Both use Sugeno and Choquet rules. The two Fuzzy rules are calculated simultaneously, and the higher result among the two is the output of the classifier. This is essentially due to the evolutionary architecture. Given the fact that the measurement is performed by means of portable cameras, in lieu of fixed systems with the eventual instrumentation as reported in the introduction, the use of EFI makes quick computation easier.

## 5. Results and Discussion

**Figure 10:**
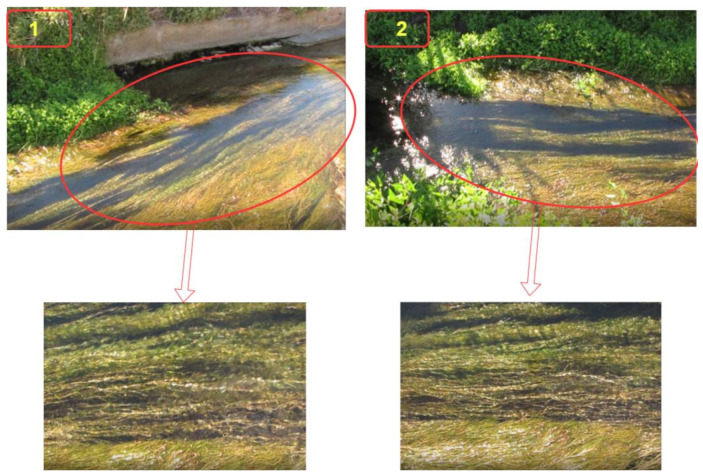
Single frames taken from the video sequences and their corresponding regions of interest.

The results reported here account for both approaches, that is, particle tracking to characterize the hydrodynamic regime and extract the velocity profiles, as well as the related metrics for targeting the region of interest. Figure 10 shows two examples of frames taken from the recorded video sequences. The regions of interest are surrounded with red ellipses. All of the videos were recorded under the same conditions, namely, frame rate, shutter speed, white balance, and aperture. By zooming in on the region of interest, one can observe a considerable grass mass moving below the water surface and hindering the free water flow.

**Figure 11:**
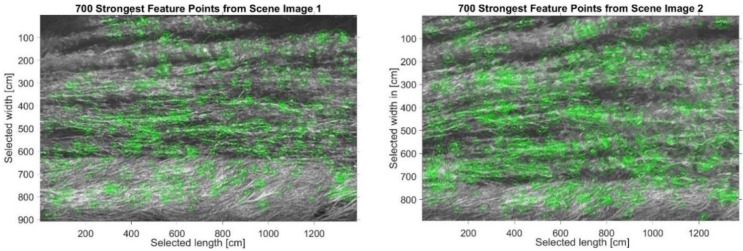
Detection of interesting regions of interest with major features.

**Figure 12:**
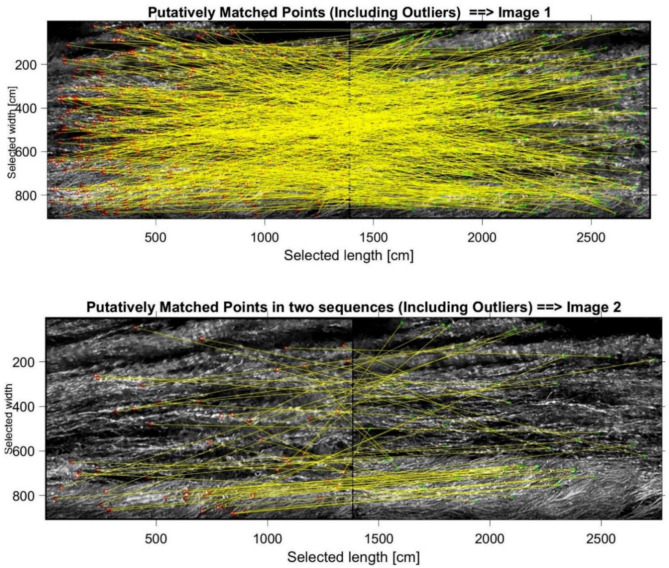
Retrieved points assumed to match the regions of interest in images 1 (**top**) and 2 (**bottom**).

**Figure 13:**
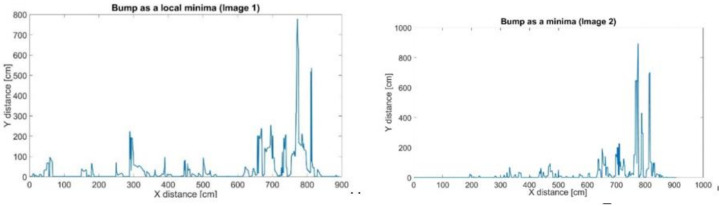
Computation of local minima for image 1 (**left**) and image 2 (**right**).

As shown in Figure 11, the algorithm is able to detect the regions of interest (ROI) in both images. The ROI is used to track the particle movement and to reconstruct a field of velocity. A major concentration of ROIs makes it easier to retrieve the velocity. Figure 12 and Figure 13 show the process of assigning tracks in the current frame to existing tracks by minimizing cost. In practice, the cost is defined here as the negative log-likelihood of the detection corresponding to a track. We started by computing the cost of assigning every detection to each track using the distance between the predicted centroid of the track and the centroid of the detection. The results were stored in a M×N matrix, where *M* is the number of tracks and *N* is the number of detections. It is important to note here that, when the motion of an object (whether it is grass of a different particle in the water) significantly deviates from this model, it may produce tracking errors. These errors can be reduced by using more complex models, such as constant acceleration or multiple Kalman filters for each tracking object.

**Figure 14:**
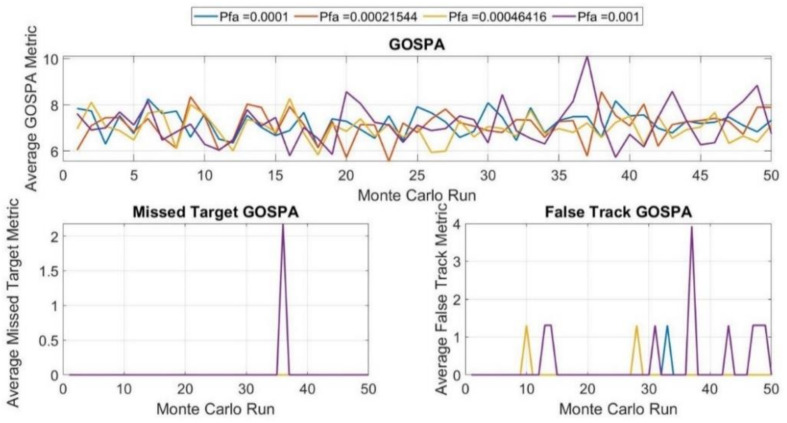
Metrics used to evaluate the computation results for all of the scenes.

Figure 14 shows the performance of the tracker in a scenario based on running 50 Monte Carlo realizations for each possible false alarm rate. Any rise in false alarm rate, for example when Pfa = 0.0001, Pfa = 0.0002, and Pfa = 0.001, corresponds to an increased probability of generating false tracks. This probability is much higher in proximity to the camera, where the density of the resolution cells is much higher. Therefore, as false alarms are closely spaced and appear frequently in this region, they can be misclassified as low-velocity false-tracks. This tracker behavior can be observed in the average false tracker component of the GOSPA metric per scenario run. It is worth mentioning here that, as the false alarm rate increases, the number of peaks in the plot increases as well. This also leads to an increase in the total GOSPA metric. The missed target component is zero for most runs.

**Figure 15:**
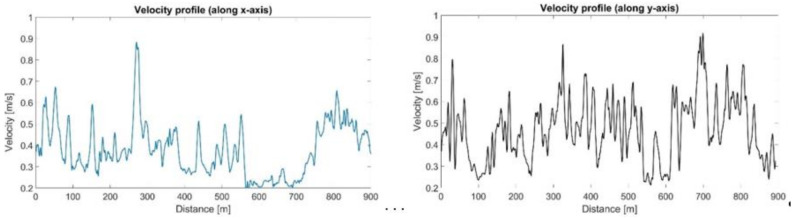
Cross and longitudinal velocity profiles.

The recovered velocity profiles can be broken down into two components, the cross and longitudinal velocity profiles, as shown in Figure 15. The maximum values of both components are around 0.9 m/s, compatible with the flow rate reported in the previous section.

**Figure 16:**
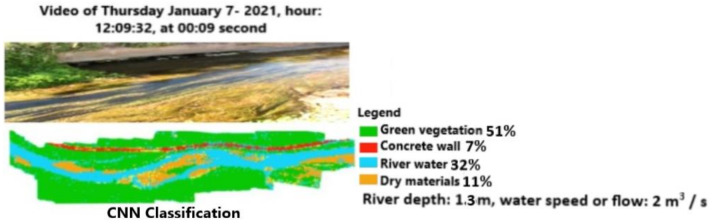
Video frame classification based on a CNN at 12:09:32.

**Figure 17:**
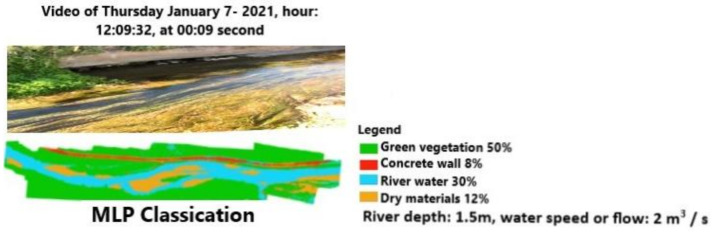
Video frame classification based on an MLP at 12:09:32.

The machine learning approach, using the powerful tool connected to the classification of all video frames, delivers more details than the previous one. Figure 16 and Figure 17 show, for the same time range of Figure 10, how the CNN and MLP classifications can recognize the main elements of the scene, such as the green vegetation, the concrete wall along the channel, the surface of water, dry materials, and river depth. The values in the legend can be verified by processing a corresponding satellite image. In particular, the channel depth (1.3 m) and the flow (2 m^3^/s) are close to the measured values, as reported in Section 2. The difference in the amount of vegetation or water surface classified by CNN and MLP is <1%.

**Figure 18:**
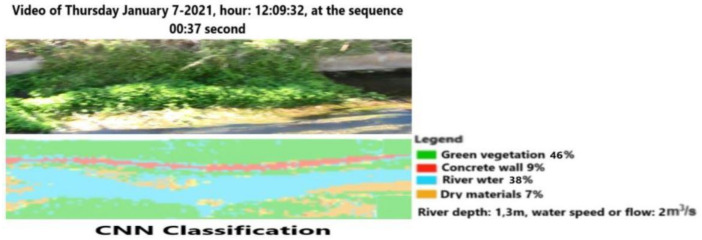
Video frame classification based on normal CNN-evolutionary fuzzy integral (EFI) at 12:09:32 and the successive frame.

**Figure 19:**
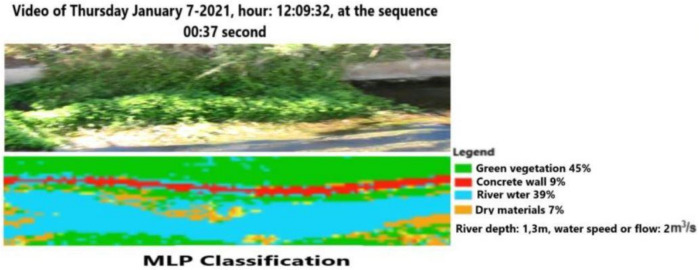
Video frame classification based on MLP at 12:09:32 and the successive frame.

The same process is repeated for the same video using a later video frame (00:37 s elapsed time) at the same position of the previous figures. Figure 18 shows the results of the CNN-EFI classification using a different angle of view. The integration of the EFI algorithm allows for an increase in the accuracy of segmentation. In fact, the amount of water surface detected here is 38% instead of 32% of the traditional CNN, while the dry materials account for 7% of the scene instead 11%. We wish to note that dry materials, such as stones, are located on the bed of the channel. Figure 19 reports classification based on MLP without further algorithm modifications. The advantage of this approach consists of the possibility of extracting further information, namely channel depth and water flow values.

The results included in this section intend to demonstrate the use of accurate and low computationally cost algorithms to monitor the flow of an artificial channel, serving mainly to collect water coming from springs and to [23,24] spill it into the sea. They fall within the hydrodynamics characterization of the channel with video-sensors and image-sensors [25].

## 6. Conclusions

The suite of the proposed algorithms is composed of an improved particle tracking algorithm and a CNN with MLP to globally monitor the cross and the longitudinal velocities of water. To better understand the results of the particle tracking algorithm, the accuracy of the tracker was determined with the generalized optimal subpattern assignment (GOSPA). The calculated longitudinal velocity is in good agreement with the history of the channel, as reported in Section II. Conversely, for the classification of the main features of the acquired video sequences, the CNN and MLP work on major data availability and trace out complementary features, namely the composition of different items, and the channel depth, as well as the magnitude of the flow. This is close to the value detected by the particle tracking algorithm and by the measurement history reported in Section II. The proposed suite of tools can be easily implemented using a smartphone as an image sensor, as these devices are capable of producing excellent results even when used as fixed monitoring stations.

The great interest in hydrodynamic monitoring is constrained by two main aspects: the flow level and volume of water, as well as the computational time to exhibit results. As reported in the updated introduction, and in updated sub Section 4.2, in case of channels located in areas where there are difficulties and reasons that prevent the installation of dedicated instrumentation, the approach carried out in this paper can be of help as it is applicable on video-recordings and fixed frames of images. It allows for the use one or more portable devices for capturing video-recordings and images, as well as a laptop to process the aforementioned data for displaying the envisaged results. Using an I7 processor with 32 GB RAM, the particle tracking-based algorithm displays the output in 19 min, and the machine learning results are obtained after 4 min. Combining both algorithms, we are able to know the field of velocity within the flow, that is a propagation of different particles, and this is very important in the presence of polluted water with reference to, for instance, hydrocarbons, for which the field of velocity is necessary in order to know if they are deposited on the sides of the channel or on the banks of a river. Knowing this, we can correctly sample materials for further chemical and physical investigations. Conversely, the artificial network-based algorithms using separately CNN and MLP allow for either retrieving the flow velocity or the nature of the materials constituting the channel under test. The complex of the proposed suites is an innovating way to characterize a channel. It can be also remotely done using satellite images.

## Data Availability

Not applicable.
